# Effects of biodegradation of starch-nanocellulose films incorporated with black tea extract on soil quality

**DOI:** 10.1038/s41598-024-69841-2

**Published:** 2024-08-13

**Authors:** Elham Malekzadeh, Aliasghar Tatari, Mohammadreza Dehghani Firouzabadi

**Affiliations:** 1 Department of Soil Science, Gorgan University of Agricultural Sciences and Natural Resources, Basij Square, PO Box: 4918943464, Gorgan, Golestan Iran; 2 Department of Paper Science and Engineering, Gorgan University of Agricultural Sciences and Natural Resources, Gorgan, Iran

**Keywords:** Biodegradation, Film, Nanocellulose, Soil properties, Starch, Tea extract, Biochemistry, Biogeochemistry, Environmental sciences, Engineering

## Abstract

This study aimed to investigate the biodegradation behaviour of starch/nanocellulose/black tea extract (SNBTE) films in a 30-day soil burial test. The SNBTE films were prepared by mixing commercial starch, nanocellulose (2, 4, and 6%), and an aqueous solution of black tea extract by a simple mixing and casting process. The chemical and morphological properties of the SNBTE films before and after biodegradation were characterized using the following analytical techniques such as field emission scanning electron microscopy (FESEM), energy-dispersive X-ray spectroscopy (EDX), and fourier transform infrared (FTIR). The changes in soil composition, namely pH, electrical conductivity (EC), moisture content, water holding capacity (WHC), soil respiration, total nitrogen, weight mean diameter (MDW), and geometric mean diameter (GMD), as a result of the biodegradation process, were also estimated. The results showed that the films exhibited considerable biodegradability (35–67%) within 30 days while increasing soil nutrients. The addition of black tea extract reduced the biodegradation rate due to its polyphenol content, which likely resulted in a reduction in microbial activity. The addition of nanocellulose (2–6% weight of starch) increased the tensile strength, but decreased the elongation at break of the films. These results suggest that starch nanocellulose and SNBTE films are not only biodegradable under soil conditions but also positively contribute to soil health, highlighting their potential as an environmentally friendly alternative to traditional plastic films in the packaging industry.

## Introduction

In biodegradable packaging, biopolymers such as starch, chitosan, and cellulose are obtained from renewable raw materials such as agricultural waste or food processing waste^1–3^. Starch is a complex polysaccharide found in all plant tissues. It consists of linear chains of α-d-glucose units linked by α(1–4)-glycosidic bonds. These chains also occasionally contain α(1–6) glycosidic links, which give the starch its branched structure^4,5^. The hierarchical organization of starch gives it a distinct semi-crystalline structure that influences its physical and chemical properties as well as its ability to be digested by enzymes^6^. The macromolecular nature of starch makes it an important resource in several industrial sectors such as food, pharmaceuticals, and packaging^7,8^.

Starch is a suitable candidate for the production of biodegradable films for sustainable packaging due to its renewable source, biocompatibility, and inherent biodegradability^1,2,9–11^. In packaging applications, starch can be fabricated into films with desirable mechanical properties and barrier properties by utilizing processing methodologies such as casting^9^ or extrusion^12^. Further, the need for biodegradable alternatives to plastic pollution emphasizes the need for starch-based films, which are promising contenders because of their biodegradability^13,14^. Starch-based films are biodegradable, but they are also flexible, allowing them to be tailored to specific packaging needs by incorporating additives or blending with other biopolymers^9,15–17^. Therefore, the investigation and improvement of packaging films made from starch show great potential in tackling the increasing environmental issues linked to traditional plastic packaging^18,19^.

Cellulose is the most abundant organic compound in the world^20^. Nanocellulose is characterized by the deconstruction of the hierarchical arrangement of cellulose chains at the nanoscale, resulting in a unique chemical makeup^21–23^. The cellulose chains are produced into nanocellulose by mechanical, biological, or chemical processes^24–27^. Nanocellulose exhibits both crystalline and amorphous areas^28^. Nanocellulose particles possess remarkable mechanical capabilities, characterized by their outstanding tensile strength, Young’s modulus, and flexibility^24,28,29^. These qualities are attributed to their high aspect ratios and surface areas-to-volume ratios. In addition, nanocellulose has a high level of reactivity as a result of the many hydroxyl (−OH) groups present in the cellulose chains^30^. This reactivity enables easy chemical modification and functionalization, allowing for the customization of its features to suit a wide range of applications, including the production of biodegradable packaging films.

Black tea extract, obtained from the leaves of the *Camellia sinensis* plant, is a highly abundant source of polyphenols, specifically catechins, and theaflavins, which exhibit strong antioxidant properties^31,32^. These compounds not only contribute to the characteristic flavour and colour of black tea, but also offer potential benefits for packaging films^11,17,33–36^. Incorporating tea extract into packaging films can impart antioxidant activity, extending the shelf life of packaged products by delaying oxidation and microbial degradation^11,15–17,37–42^. Furthermore, the polyphenols in black tea extract have been shown to exhibit antimicrobial properties^43^, inhibiting the growth of spoilage microorganisms and pathogens, thereby enhancing the safety and quality of packaged goods^44,45^. Moreover, the natural origin of black tea extract aligns with the growing consumer demand for sustainable and eco-friendly packaging solutions^46^. By using the antioxidant and antimicrobial properties of tea extract, packaging films can offer enhanced protection and preservation of food products while reducing the need for synthetic additives or preservatives^10,16,34,35,37,38,47,48^. Therefore, the incorporation of tea extract in packaging films represents a promising path to develop functional and sustainable packaging materials with value-added for the food industry^49,50^.

The reinforcement of biopolymer packaging films with new materials is very important. Considering the increasing importance of renewable energy and the need to replace many plastics in the packaging industry, reinforcing biopolymer films with new materials can significantly improve the physical properties and performance of these films. For example, the use of plant extracts, nanocellulose and starch derived from various plants as additives in biopolymer films can act as a natural reinforcement and improve the mechanical, tensile, and moisture-resistant properties of these films, as well as increase their stability and useful life. In addition to increasing the physical properties, these additives can also significantly improve erosion resistance, gas permeability and heat resistance, which is very important for packaging applications. On the other hand, these new materials can help to fulfill the environmental goals of the packaging industry, because they use biological and renewable resources and reduce the amount of plastic waste production. In recent years, research related to black tea extract, nanocellulose, and starch in the field of production and reinforcement of biopolymer packaging films has grown significantly. Andrade et al.^49^ investigated polylactic acid (PLA) films loaded with polyphenol extracts from green tea and rosemary. They reported that these active PLA packaging can contribute to the delay of lipid oxidation in foods with high-fat content. Carrizo et al.^50^ reported that active food packaging based on green tea extract can have antioxidant capacity compared to control (without green tea extract). Homthawornchoo et al.^38^ investigated the effects of incorporating green tea extract (GTE) into rice starch-pectin (RS-P) blend films. The addition of GTE improved the films' antioxidant properties and increased their thickness. However, it also decreased transparency, moisture content, and water vapor transmission rate (WVTR). GTE weakened the films but showed some antimicrobial activity against Staphylococcus aureus. Medina‐Jaramillo et al.^40^ investigated the use of green tea and basil extracts as natural plasticizers in cassava starch-based biofilms for food coatings. They reported that green tea and basil components interact strongly with starch molecules to increase their molecular mobility, which leads to plasticization. As a result, the films were thermally stable and retained high transparency at 250 °C. Yuan et al.^48^ investigated the development of active food packaging films using biopolymers derived from shrimp shell waste protein (SSWP) and chitosan (C). The film characteristics were enhanced by incorporating oolong tea extract (OTE), maize silk extract (CSE), and black soybean seed coat extract (BSSCE) at different concentrations (1, 3, and 5%, w/w). They found that adding OTE, CSE, and BSSCE significantly impacted the films physicochemical properties. Film thermal stability improves with increasing extract concentration. Rodrigues et al.^39^ reported the effect of incorporation of GTE, ginger essential oil (GEO), and nanofibrillated cellulose (NFC) on the properties of starch films. They concluded that reinforcing the films with nanofibrillated cellulose improved their strength, and reduced water solubility and WVTR. The natural extracts, on the other hand, increased antioxidant activity and inhibited the growth of bacteria. Rajapaksha and Shimizu^10^ found that by incorporating microencapsulated spent black tea extract (SBT), active films were improved mechanically and antioxidant. Compared to the control starch film, both types of active films were better at blocking UV light and water vapour and retarding lipid oxidation for 35 days. In a study by Perazzo et al.^16^, active films made from cassava starch bio-based and infused with aqueous green tea extract and palm oil colourants maintained butter quality for 45 days. They recommended avoiding high concentrations of green tea extract as additives to films due to its high polyphenol content, which can act as a pro-oxidant agent. A study conducted by Peng et al.^47^ found that GTE significantly reduced water vapor permeability and increased film antioxidant ability. GTE films were able to scavenge DPPH radicals more effectively than black tea extracts (BTE) films across all food simulators (0, 20, 75, and 95% EtOH).

The literature review showed that research on starch films containing tea extracts is promising, but some areas could benefit from further investigation. While studies are investigating different concentrations of tea extracts, there are insufficient and comprehensive studies on starch films containing black tea extract and nanocellulose. Further research is needed on how these films behave during prolonged storage. Some studies have looked at green or black tea extracts. A closer look at the specific bioactive components responsible for the effect could lead to more targeted film formulations. Most research has focused on the properties of starch films with tea extracts for food packaging, while the behaviour of biodegradation on soil respiration and nutrients has not been studied.

**Figure 1:**
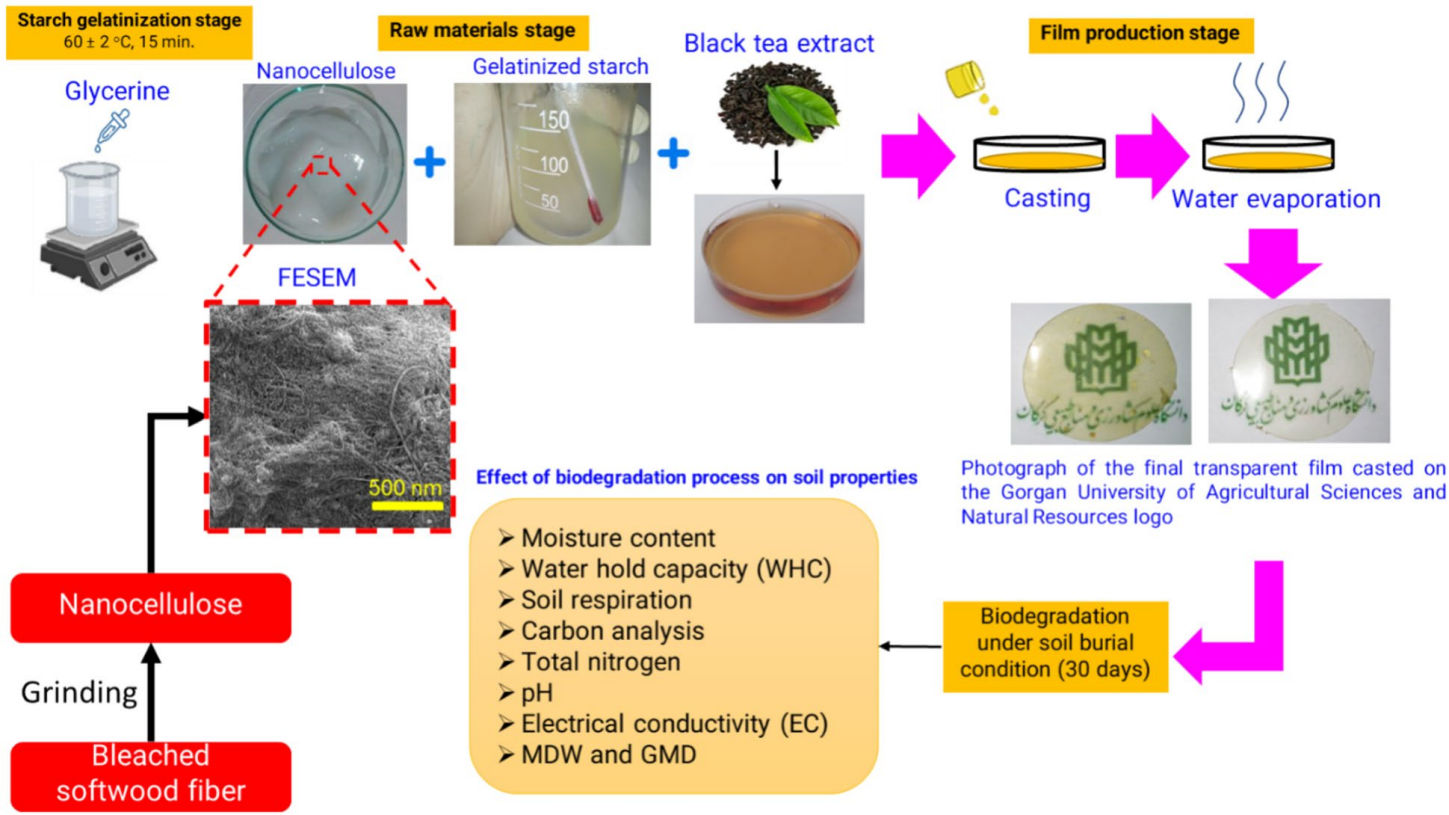
Schematic of the conceptual design of the research.

In investigating the biodegradation process of SNBTE films in soil, there may be a research gap regarding the extended effects of these films on soil microbial communities and nutrient cycling. Current research is mainly focused on the degradation rate and structural durability of these films. However, there is limited knowledge of their effects on soil microbial activity, such as respiration rates and microbial biomass, and on nutrient dynamics, such as changes in carbon, nitrogen and phosphorus availability in the soil ecosystem. Addressing this research gap would provide important insights into the ecological consequences of using these biopolymer-based films as sustainable packaging materials. It would also contribute to the development of more thorough life cycle assessments to evaluate their environmental impact. This study aims to evaluate the medium-term degradation of SNBTE films in soil and to investigate the changes in film integrity, microbial activity, and nutrient availability over time. By evaluating soil respiration rates as an indicator of microbial activity and monitoring changes in soil nutrient content, including carbon (C), nitrogen (N), and phosphorus (P), this study will investigate the interactions between biodegradable films and soil ecosystems (Fig. 1). These findings can be used to improve packaging design and use in sustainable applications by providing a better understanding of these materials' environmental behaviour.

## Material and methods

### Materials

Corn starch (72 wt.% amylopectin and 28 wt.% amylose) and glycerol as a plasticizer (food grade, purity 99%) were purchased by Merck (Darmstadt, Germany) and used without further purification. Unblended and dried black tea was obtained from a local market in Ashkevarat city, Gilan province. Distilled water was used in all experiments.

### Preparation of black tea extract (BTE)

The aqueous BTE was prepared following the previous report, with slight modifications^10,33,38–40,51^. In brief, aqueous extract was obtained by dispersing 5.0 g of dried black tea in 100 mL of distilled water at 100 °C for 40 min. Immediately, the hot extract was cooled to room temperature and filtered twice (Whatman No.1 filter paper). The solution was kept in dark containers at ~ 5 °C until further use.

### Preparation of nanocellulose

The nanocellulose gel (2.5 wt%) was prepared from commercially bleached softwood pulp. To produce nanocellulose, water slurry containing cellulose fibres at 1.0 wt% was passed three times through a disk grinder (MKCA6-3; Masuko Sangyo Co., Ltd., Japan)^52–54^. To concentrate the gel, a Sigma KS centrifuge apparatus (Sigma Co., Germany) was used at 14,000 rpm for 30 min^26^.

### Preparation of films (casting method)

Corn starch powder (5.0 g) and glycerol as plasticizer (1.5 g) were dissolved in 100 mL distilled water with constant stirring (65 ± 2 °C for 15 min) to achieve complete gelatinization^9^. To prepare SNBTE films, three different concentrations of nanocellulose (2, 4, and 6 wt% of starch) were added to a partially gelatinized starch solution with stirring to ensure uniform dispersion of nanocellulose in the film matrix. In addition, 2 wt% BTE was incorporated into the film solution while maintaining stirring at 65 °C for 15 min. For film formation, 25 g of each gel was poured onto 10 cm plastic Petri dishes and then immediately dried in a drying oven at 60 °C for 8 h. After drying, the films were conditioned at 23 °C and 50% relative humidity (RH) for at least 48 h before characterization.

### Characterization

#### Mechanical properties

The tensile properties were determined based on the American Society for Testing and Materials (ASTM) D882-18 standard, using a SANTAM universal tensile machine (model STM-1, Santam Co., Tehran, Iran) with a load cell of 1 kN and cross-head speed 10 mm/min. Film samples were cut in dumbbell shapes and mounted between the tensile grips. Three samples from each film were conditioned at temperature = 23 °C and RH = 50% for at least 48 h before testing. Tensile parameters including tensile strength and elongation at break were reported.

### Morphology (FESEM/EDX analysis)

The surface morphology of the produced films was examined using field emission scanning electron microscopy (FESEM, MIRA3 TESCAN-XMU, Kohoutovice, Czech Republic) and energy-dispersive X-ray analysis (EDX, SAMx Numerix, Levens, France). The samples were dried at 40 °C for 12 h and mounted on aluminum stubs using double-sided carbon tape. Then, samples were coated with gold using a sputter coating machine (Quorum, Q150R ES, UK). The micrographs were captured at an accelerating voltage of 10.0 kV.

### X-ray diffraction (XRD)

XRD is an extensively employed technique for characterizing various substances and determining their crystallinity. The X-ray characterization was performed using a diffractometer XRD (Unisantis XMD300 model, Singapore) equipped with monochromatic Cu-Kα radiation at 50 kV, 30 mA, and a sampling width of 0.02 degree. Scan values varied between 10 and 60 (2 theta).

### Fourier transform infrared (FTIR) spectroscopy

The FTIR is a technique used to analyze the chemical composition of materials and identify and quantify different compounds by measuring the absorption of infrared light. FTIR spectrometers (Perkin-Elmer, Spectrum RX I) were used to examine the functional groups and chemical changes of films. A total of 64 scans were acquired with a resolution of 4 cm^−1^, covering the range from 4000 to 500 cm^−1^.

### Water hold capacity (WHC) and moisture content

The WHC of soil was determined according to Tai et al.^55^ study. Briefly, the soil was mixed with 20.0 g of distilled water, and the excess water was drained. The soil was subsequently saturated and deposited on filter paper that had been pre-dried to a constant weight. The saturated weight (W_sat_) was then recorded. A dry weight (W_dry_) was determined by drying the sample at 105 °C until it reached a constant mass, and the WHC was calculated by using Eq. (1). The initial weight (W_ini_) of each soil sample was recorded on a previously dried filter paper. Subsequently, the samples were desiccated in an oven set at 105 °C until their weight remained constant, yielding the final soil mass (W_fin_). The soil's moisture content was determined utilizing Eq. (2).

1 $$\text{WHC }(\text{\%})\hspace{0.17em}=\hspace{0.17em}\frac{{\text{W}}_{sat}- {\text{W}}_{dry}}{{\text{W}}_{sat}}\times 100$$

2 $$\text{Moisture content }(\text{\%})\hspace{0.17em}=\hspace{0.17em}\frac{{\text{W}}_{ini}- {\text{W}}_{fin}}{{\text{W}}_{ini}}\times 100$$

### Soil respiration

In soil respiration, carbon dioxide (CO_2_) is produced as a result of metabolic processes. As a consequence, soil respiration represents biological activity. With the excess sodium hydroxide (NaOH) titrating technique, soil respiration was measured every 10 days for 30 days^9^.

### Biodegradability test

The biodegradability of films was evaluated using a slightly modified version of previously established techniques for soil burial^9,56,57^. The physical and chemical properties of the soil were determined as follows: EC (0.34 ds/m), pH (7.10), total organic carbon (0.35%), total nitrogen (0.014%), saturation point (44.0%), field capacity (35.32%), MDW (0.02 mm), GMD (1.03 mm), and texture (silty-clay-loam). To test the biodegradability of the films, 100 g of soil was mixed into a 250 mL beaker. Samples were cut (40 mm^2^) and buried in the soil mixture in the beaker. The soil moisture was consistently kept at 27 °C and 70% of field capacity (70% FC). Dry samples were buried for 10–30 days in the wet soil of the test medium. Visual changes were occasionally observed in these samples. Following the removal of the film samples from wet soil after 10–30 days, they were repeatedly rinsed with water to remove soil adhering to the surfaces. In biological degradation testing, films were dried at 60 °C for 2 h until their weight became constant. The weight loss was calculated by using Eq. (3).

3 $$\text{Weight loss }(\text{\%})\hspace{0.17em}=\hspace{0.17em}\frac{\text{Initial weight}-\text{ Final weight}}{\text{Initial weight}}\times 100$$

### Carbon analysis

In this study, the contents of total organic carbon (TOC), cold water extractable organic carbon (CWEOC), and hot water extractable organic carbon (HWEOC) were determined. TOC was measured using both the original and modified Walkley–Black (WB) dichromate method^58^. The CWEOC and HWEOC determining was performed according to the method described in the Hamkalo and Bedernichek^59^ report.

### Total nitrogen

The total nitrogen (N) content of the soil was determined using the Kjeldahl method, a widely recognized and reliable technique for measuring nitrogen levels in various samples. This method involves digesting the soil sample with concentrated sulfuric acid and a catalyst, which converts organic N into ammonium ions. The ammonium ions are transformed into ammonia gas by heating and distillation. The ammonia is then titrated with a standardized acid solution to determine the N content of the sample^60^.

### pH and electrical conductivity (EC)

Soil pH was assessed with a digital pH meter (model PH700 Benchtop pH Meter). The electrical conductivity (EC) of the soil was evaluated using an EC meter (Hanna Instruments, Model HI5321-02) on an aqueous soil extract.

### Mean weight diameter (MDW) and geometric mean diameter (GMD)

Aggregate stability was measured according to MWD using Eq. (4)^61^, and geometric mean diameter (GMD) was measured using Eq. (5)^62^.

4 $$MDW\, = \,\sum\limits_{i = }^n {\overline {Xi} } Wi$$

where, $\overline {Xi}$ is the mean diameter of aggregates for any particular size range; W_i_ is the weight of the aggregates in a size range as a fraction of the total dry weight of the sample; and n is the number of sieves.

5 $$GMD = \exp \frac{{\sum\limits_{i = 1}^n {WiLog\overline {Xi} } }}{{\sum\limits_{i = 1}^n {Wi} }}$$

where, W_i_ is the weight of the aggregates in a size range of average diameter Xi; and ∑ is the total weight of the soil sample.

### Statistical analysis

The data obtained from the investigations underwent analysis of variance (ANOVA) utilizing the SPSS 24.0 software (IBM, Armonk, NY, United States; https://www.ibm.com).

## Results and discussion

### Nanocellulose characterization

**Figure 2:**
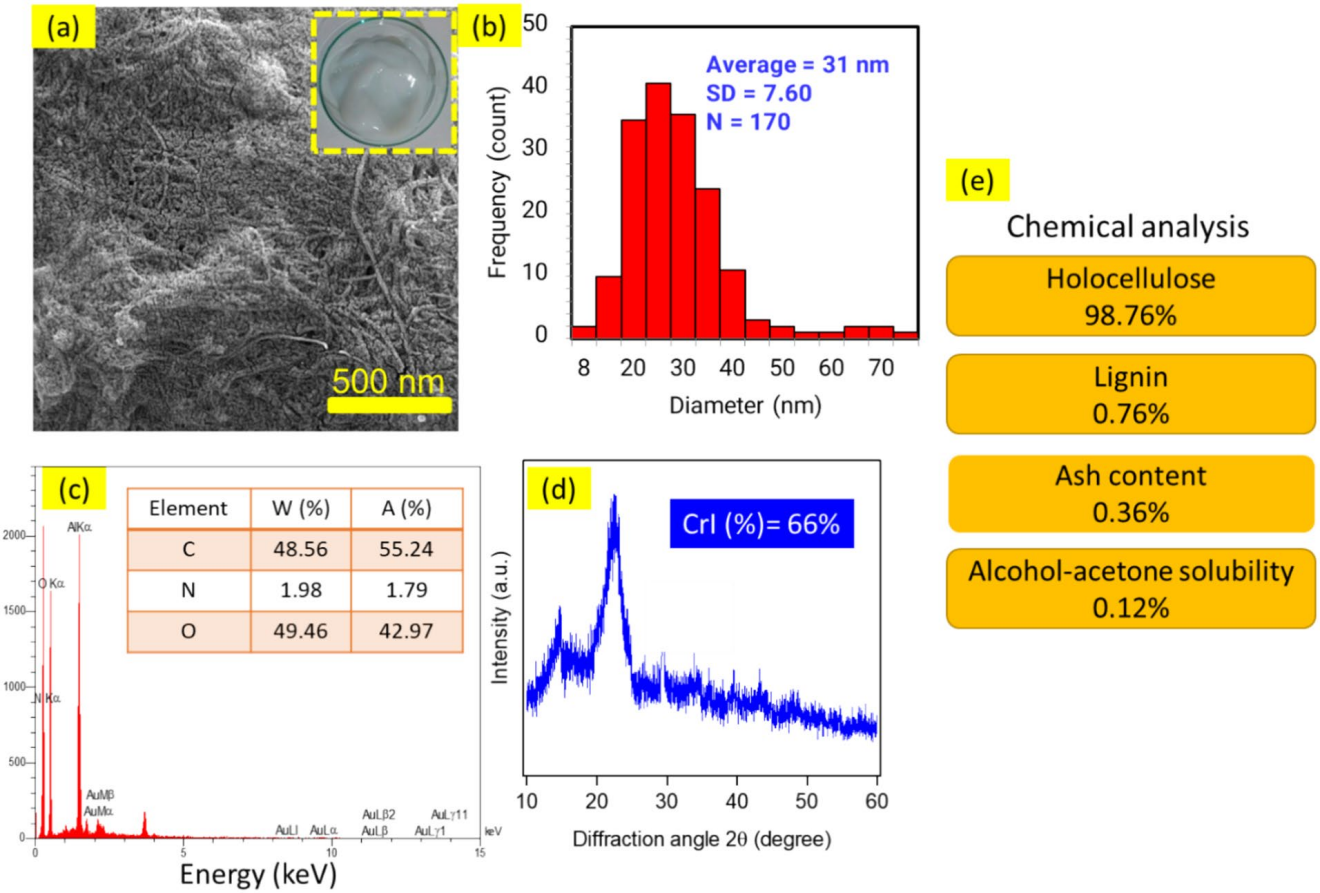
Morphological and chemical characteristics of nanocellulose used in this research: (**a**) FESEM micrographs, (**b**) diameter distribution histogram, (**c**) EDX spectrum, (**d**) XRD diffraction pattern, and (**e**) chemical analysis.

The physicochemical and morphological properties of the nanocellulose used in this study, including FESEM micrographs, diameter distribution histogram, EDX analysis, XRD diffraction pattern, and chemical analysis, are presented in Fig. 2. The diameter of the nanofibers was determined by analyzing FESEM micrographs (N = 170) with Digimizer image analysis software (version 4.1.1.0; MedCalc Software, Mariakerke, Belgium; http://www.digimizer.com), revealing a fibrillar structure with an average diameter of 31 ± 7.60 nm (Fig. 2a,b). The diameter of the nanocellulose used in this study was similar to other nanofibers obtained from different sources, such as softwood fibre^9,63^, canola straw^28^, sugarcane bagasse fibre^52^, and waste paper industry^64^. To further understand the nanocellulose properties, the localized elemental information of nanocellulose was determined by EDX as exhibited in Fig. 2c, which contains intense signals of C, N, and O. The XRD pattern of the as-prepared nanocellulose is presented in Fig. 2d. The absence of significant diffraction peaks in the whole pattern indicates that the nanocellulose generated is predominantly crystalline. The peaks were observed at 2ϴ of 15.1, 16.2, 22.4, and 34.1 degrees, indicating the presence of cellulose type Iβ^65^. The chemical composition values of nanocellulose used in this study are provided in Fig. 2e. The high amount of holocellulose and the low amount of lignin indicate the confirmation of the quality of the raw materials for the production of nanocellulose. Also, the amount of less ash content indicates the minimum mineral impurities in the produced nanocellulose.

### Mechanical properties

The results for the mechanical properties of the starch/nanocellulose/black tea films are shown in Table 1. The addition of the tea extract decreased the values for tensile strength and elongation at break compared to the films without extracts. The results showed a significant increase in the tensile strength of the films with the addition of nanocellulose. The maximum tensile strength was achieved when 6.0 wt% nanocellulose was incorporated into the SNBTE films. The tensile strength was increased to 10.54 MPa, an improvement of 40.4% compared to the control. This result was supported by Ali et al.^66^ who found that the addition of nanocellulose from sugarcane bagasse increased the tensile strength but decreased the elongation at the break of PVA/starch composite films with nanocellulose. Othman et al.^67^ reported that the addition of 1.5% nanocellulose (in weight percent of starch) increased the tensile strength but decreased the elongation at the break of corn starch films. Hafid et al.^68^ found that the use of surface-modified cellulose from glossy paper waste in starch-based films increased tensile strength.

**Table 1:** Mechanical properties of starch/nanocellulose films incorporated with tea extract.

Film	Tensile strength (MPa)	Elongation at break (%)
Control (pure starch)	7.51 ± 0.72	37 ± 3.0
Starch-2% nanocellulose	9.33 ± 0.86	27 ± 3.0
Starch-4% nanocellulose	9.76 ± 0.73	20.41 ± 1.80
Starch-6% nanocellulose	10.54 ± 0.87	12.51 ± 1.60
Starch-2% nanocellulose-tea extract	8.45 ± 0.69	17.54 ± 1.33
Starch-4% nanocellulose-tea extract	9.23 ± 0.62	14.52 ± 1.47
Starch-6% nanocellulose-tea extract	9.66 ± 0.66	10.06 ± 1.23

Values represent the mean ± standard deviation.

The cellulose-based nanomaterials possess a large specific surface area and exhibit good dispersion. These characteristics enhance the interfacial bonding between the filler and matrix, facilitating the efficient transfer of stresses from the matrix to the particles. As a result, the mechanical properties of the film are improved^69^. Because the cellulose fibers in the films were nanosized and could effectively make contact with the corn starch matrix, they promoted high intermolecular forces, increasing the tensile strength of the films containing nanocellulose. These forces led to an increase in the stiffness of the films and thus to a high tensile strength. In addition, the entanglement of the nanocellulose within the starch polymer chains may have contributed to the increase in tensile strength^66,67^. The strong interactions between the matrix and filler prevent the SNBTE films from elongation.

The decrease in tensile strength and elongation at break in starch/nanocellulose films incorporated with black tea extract is primarily attributed to the disruptive effects of the tea extract on the intermolecular interactions within the film matrix. The incorporation of tea extract alters the structural integrity of the film, possibly through interactions with bioactive compounds such as polyphenols and antioxidants contained in the extract. These interactions can lead to changes in the morphology and composition of the film, ultimately affecting its mechanical properties. In addition, the presence of tea extract can lead to new interactions between the film components that further influence the overall mechanical behaviour of the film. Rodrigues et al.^39^ found that reinforcement with nanocellulose (2%) resulted in a significant increase in tensile strength (47.6%) and lower elongation compared to the control (starch films). Peng, et al.^47^ reported that the use of green tea and black tea extract in chitosan packaging films decreased tensile strength and elongation at break. Similar results have been found in PLA and starch films incorporated with rosemary extracts^49,70^ and thymol^67^. Martins et al.^15^ assessed the tensile strength in the longitudinal direction of an active PLA film enhanced with green tea extract. They produced a control PLA film exhibiting a higher tensile strength of 40.2 MPa, which was reduced to 35.4 MPa upon incorporating 2% green tea extract.

### Biodegradability

**Figure 3:**
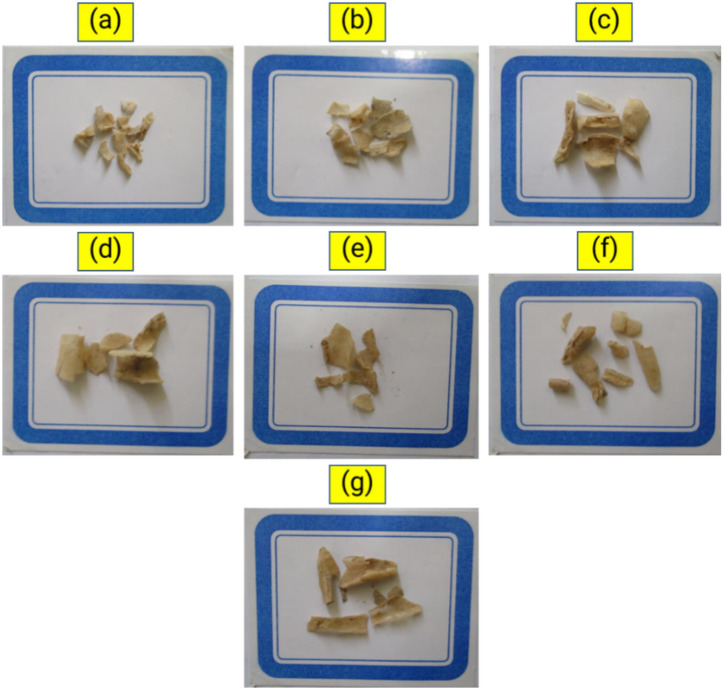
Photographs of film subjected to biodegradation in soil for 30 days. (**a**) control (pure starch), (**b**) starch-2% nanocellulose, (**c**) starch-4% nanocellulose, (**d**) starch-6% nanocellulose, (**e**), starch-2% nanocellulose-tea extract, (**f**), starch-4% nanocellulose-tea extract, and (**g**) starch-6% nanocellulose-tea extract.

In the evaluation of film degradation in soil, the biodegradable properties of the films were examined when they were buried. The percentage of biodegradation was determined using Eq. (3) and the resulting data is shown in Table 2. After 30 days in the soil, the control film lost 66.66% of its weight. The addition of nanocellulose and tea extract significantly (p < 0.05) reduced the amount of biodegradability of the films in the soil compared to the control. The addition of 6% nanocellulose to the starch films reduced the biodegradability by 41.67% compared to the control treatment. When films were treated with tea extract (starch-6% nanocellulose-tea extract), this value was 47.22% compared to the control treatment. The appearance changes of the films after 30 days of burial in soil are presented in Fig. 3.

**Table 2:** Biodegradation of films after 10, 20, and 30 days of exposure in soil.

Film	Biodegradation test time (days)		
	10	20	30
Control (pure starch)	44.44 ± 5.55	55.67 ± 1.56	66.66 ± 5.55
Starch-2% nanocellulose	31.96 ± 6.04	46.66 ± 2.22	51.85 ± 6.42
Starch-4% nanocellulose	28.29 ± 0.59	36.10 ± 2.78	44.44 ± 5.55
Starch-6% nanocellulose	24.07 ± 3.21	30.66 ± 2.78	38.88 ± 5.55
Starch-2% nanocellulose-tea extract	27.78 ± 1.11	36.07 ± 2.78	42.59 ± 3.20
Starch-4% nanocellulose-tea extract	27.07 ± 0.79	33.32 ± 1.55	42.18 ± 2.89
Starch-6% nanocellulose-tea extract	23.74 ± 8.86	30.55 ± 2.77	35.18 ± 3.21

Values represent the mean ± standard deviation.

The change in the appearance of the film was due to the amount of starch and glycerol in the formulation^57^. The decrease in the percentage of biodegradability of starch-nanocellulose films compared to control films (starch-only films) can be attributed to several interrelated factors. Firstly, the incorporation of nanocellulose into the film matrix alters its physical and mechanical properties, potentially resulting in reduced accessibility of microorganisms and enzymes responsible for biodegradation. Nanocellulose, owing to its high aspect ratio and surface area, can form a reinforcing network within the film, leading to increased crystallinity and reduced porosity, which may impede the diffusion of water and microbial degradation agents. Additionally, the compatibility between starch and nanocellulose phases, as well as the dispersion of nanocellulose within the matrix, can influence the overall degradation process. Chemical interactions between starch and nanocellulose may result in the formation of intermolecular bonds that render the composite film less susceptible to enzymatic attack and microbial degradation compared to pure starch films. Malekzadeh, et al.^9^ reported that the starch films reinforced with nanocellulose and nano lignocellulose compared to the control sample (pure starch) had lower weight loss under soil burial conditions. Trujillo-Hernández et al.^57^ reported that starch films underwent degradation, with an 83.03% weight loss observed after 15 days of exposure to soil. The samples containing 0.05% and 1% coconut bagasse cellulose had a weight loss of 82.85% and 83.65%, respectively at the same time and the samples needed more time for complete degradation in the soil compared to control. Zhang et al.^71^ stated that regenerated cellulose films underwent complete decomposition into CO_2_ and water by soil microorganisms within 2 months, with reported half-lives ranging from 30 to 42 days. Following 16 days of decay, the soil microorganisms reduced the film's molecular weight by 38–40%, accompanied by a 10–15% weight loss.

The decrease in percentage biodegradability observed in starch-nanocellulose films incorporated with tea extract compared to the control films and the starch nanocellulose films can be attributed to several factors. First, the addition of tea extract may alter the structural integrity and composition of the film matrix, potentially hindering the accessibility of microorganisms and enzymes responsible for biodegradation. Tea extract contains polyphenolic compounds that are known for their antioxidant properties and may act as inhibitors of microbial activity by scavenging free radicals and interfering with the enzymatic reactions involved in the degradation processes. In addition, the presence of tea extract can alter the balance between hydrophilicity and hydrophobicity of the film surface, affecting microbial adhesion and colonization and thus hindering the kinetics of biodegradation. Furthermore, the chemical interactions between tea extract components and film constituents could lead to the formation of more stable complexes, rendering the films less susceptible to enzymatic attack and microbial degradation. Similar results have been obtained by previous researchers^9,55,57^.

### Morphological studies

**Figure 4:**
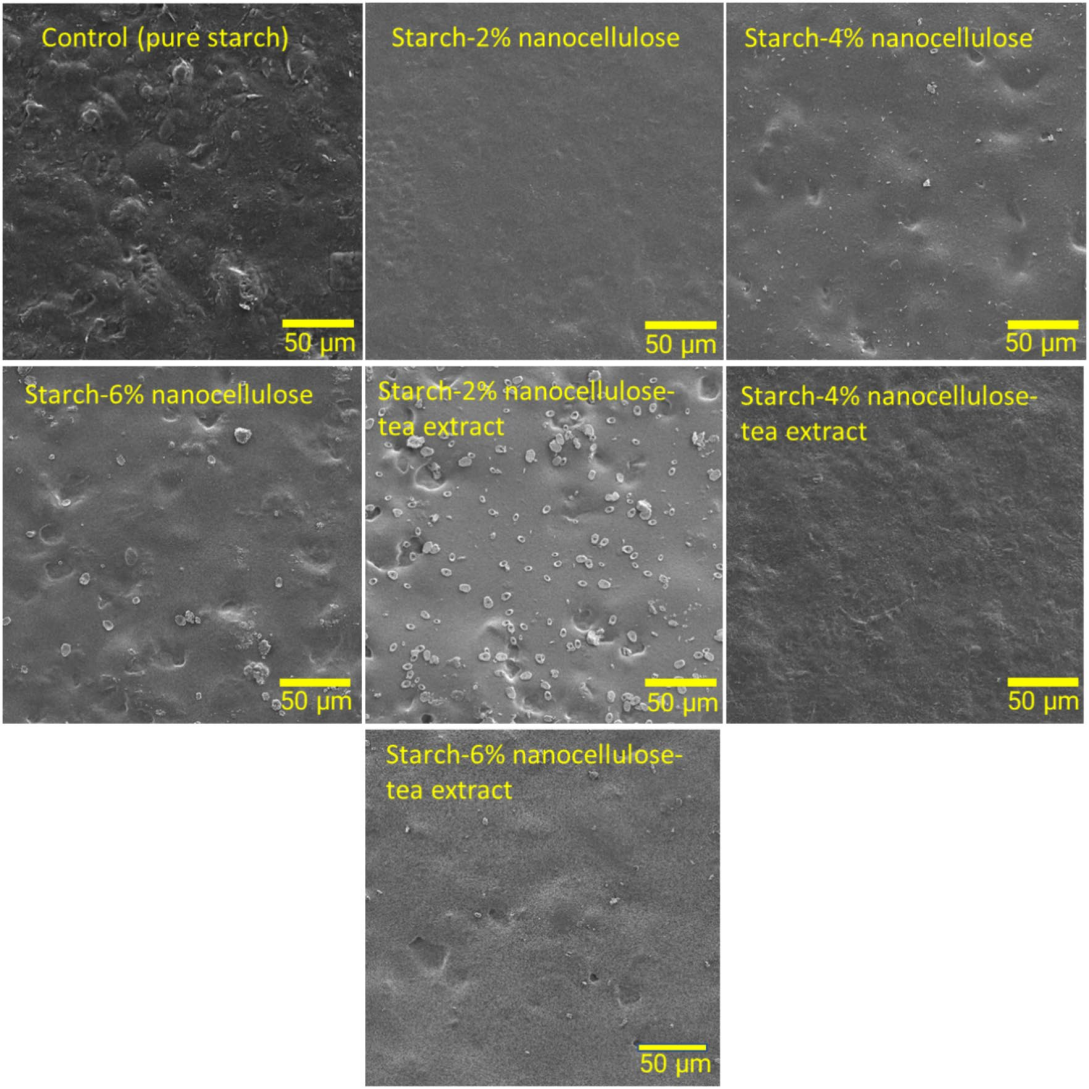
FE-SEM images of the film surface before biodegradation.

**Figure 5:**
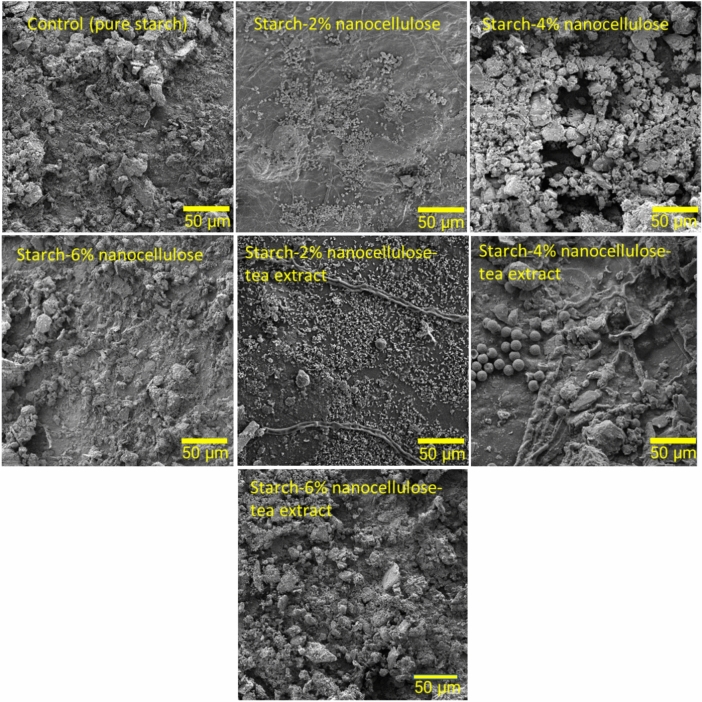
FE-SEM images of the film surface after biodegradation under soil burial conditions.

The FESEM micrographs of film samples before and after biodegradability in soil are presented in Figs. 4 and 5, respectively. The control films exhibited a notably smooth and flat surface morphology, with no detectable presence of starch particles (Fig. 4). The FESEM micrographs after biodegradation under soil burial conditions showed degradation on the surface of the film. The observed morphological shift occurred on the 30th day under soil burial conditions. The control film displayed the most extensive biodegradation, undergoing complete fragmentation. This led to the formation of significant holes and cracks on the polymer surface, evident throughout the biodegradation process. These changes are attributed to microbial activity or biofilm formation on the polymer surface, along with the diffusion of cells into the polymer's core, resulting in surface erosion and the formation of cavities within the films. The micrograph illustrates the presence of fungal spores and mycelia invading the surface (Fig. 5).

The fractures and pores observed on the surface in the films are similar to those reported by Trujillo-Hernández et al.^57^, in a film composed of starch, glycerol, and cellulose, which were degraded in 15 days. Also, similar results have been reported by Kalita et al.^72^ regarding the formation of holes and cracks in Poly(lactic acid) (PLA)-based green biocomposites surface. Batista et al.^73^ reported many changes (corrosion, cracks, and discoloration) after 2 months of biodegradation of poly(3-hydroxybutyrate-co-3-hydroxyvalerate) (PHBV)/peach palm particles biocomposites. The weak adhesion between the fiber/PHBV interface, leading to increased water absorption and enhanced accessibility for soil microorganisms, was proposed as a contributing factor to the deterioration. In a study, Zhang et al.^71^ observed a porous structure with fungal mycelium on the decayed surface in the FESEM images of regenerated cellulose films. They concluded that the biological degradation of the films was initiated by microorganisms and proceeded gradually over time.

**Figure 6:**
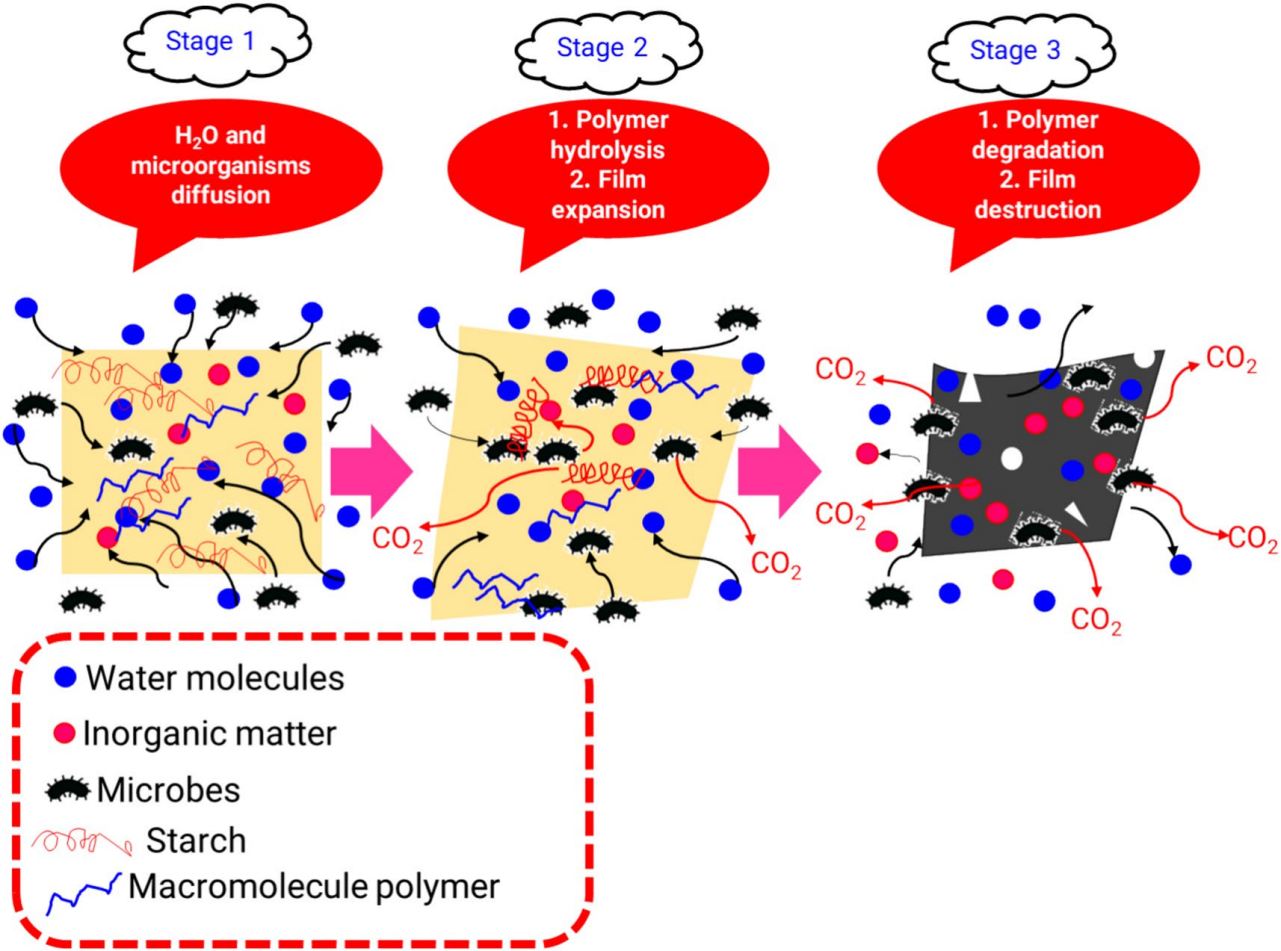
Schematic diagram illustrating mechanisms and degradation behavior of the starch-based films under soil environment**.**

The degradation of the material is caused by the proliferation of microbes on both the surface and interior^57^. In the microbial degradation process, extracellular enzymes are secreted to depolymerize the biopolymers^74^. During the biodegradation process, microbes first assimilate short chains, after which they proceed to attack long crystalline chains through a chain scission mechanism^72^. Direct contact with the soil facilitates the ability of microorganisms to locate carbon sources and initiate their metabolism, resulting in the reduction of weight in biofilms^9,57^. Starch-based materials are broken down by the process of bond cleavage, which occurs as a result of biological oxidation or biological hydrolysis and produced by microbial activity. Soil burying erosion is a physical process that involves the swelling, diffusion, and dissolving of monomers^55^. Biodegradation is the process of physical decomposition where various bacteria bind to the surface of organic materials by releasing adhesive compounds. Polysaccharides and proteins are adhesive molecules that can infiltrate materials and modify their volume, size, and pore distribution, as well as their moisture content and thermal conductivity^9^. Under regulated conditions of aerobic and anaerobic environments, microbial breakdown modifies the strength and color of polymeric material. Throughout this procedure, bacteria transform the insoluble biopolymer into a soluble biopolymer. Microorganisms utilize these chemicals in their metabolic processes for microbial growth. This conditions elucidates the process of biopolymer degradation in the presence of microorganisms, highlighting the resulting by-products such as carbon dioxide (CO_2_), water, inorganic compounds, biomass, and methane (CH_4_)^74^. In a similar study, Tai et al.^55^ explained a similar mechanism for starch-polyurethane hybrid films. Figure 6, presents a summary of the biodegradation mechanisms in starch-based films under soil environment.

Enzymatic degradation drives biodegradation by catalyzing the depolymerization of polymeric chains within a matrix. This process involves hydrolase enzymes facilitating the cleavage of chemical bonds prevalent in biodegradable bioplastics, by adding water molecules. Key enzymes like cellulases and amylases target cellulose and starch polymers, respectively, while esterases and lipases act on co-polyesters, breaking ester bonds. Factors such as surface morphology, crystallinity of biomaterials, composition mixing, burial depth (influenced by microbial and environmental variations), and duration of exposure to soil collectively influence the degree of biodegradability^75^.

### FTIR analysis

**Figure 7:**
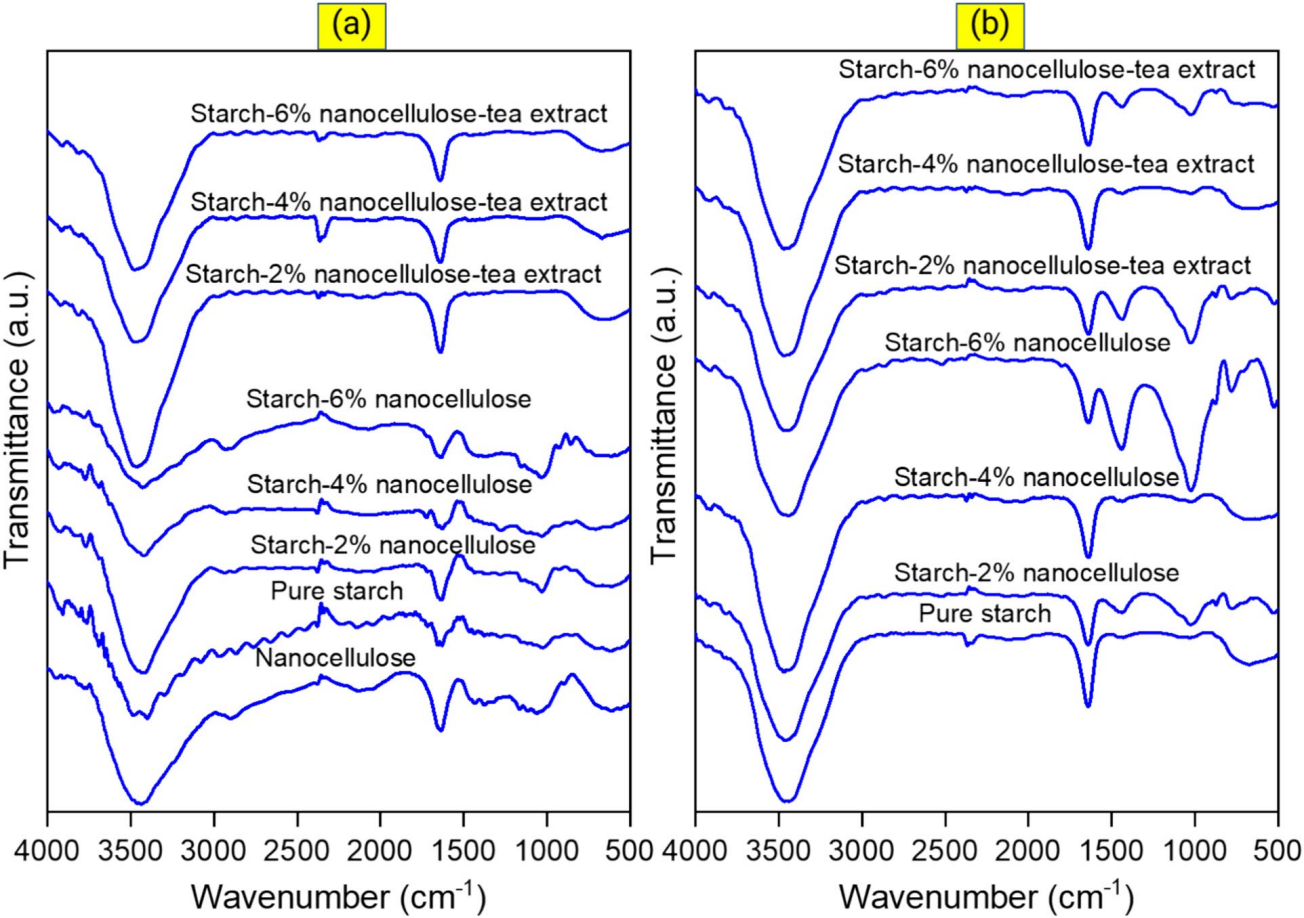
FTIR spectra of films (**a**) before and (**b**) after biodegradation under soil burial conditions (30 days).

The FTIR spectra of various films before and after biodegradation under soil burial conditions (30 days) are provided in Fig. 7. In the FTIR spectrum of pure starch, nanocellulose, starch-nanocellulose, and SNBTE films, different peaks can be recognized that indicate the chemical and structural properties of these materials. In pure starch, peaks associated with hydroxyl functional groups (–OH) and carbonyl groups (C=O) can be recognized. In pure nanocellulose, peaks consistent with the structural features of cellulose are observed, including C–O–C and C–H peaks. When nanocellulose is combined with starch, the peaks of both materials (starch and nanocellulose) can be seen, and compared to pure samples, changes in the location and intensity of these peaks can be observed, consistent with changes in the molecular structure or compounds in the example is mentioned. The addition of tea extract to starch-nanocellulose composites can lead to changes in the FTIR spectrum, including an increase or decrease in the intensity of peaks or the appearance of new peaks, which may be caused by chemical interactions between the compounds. The FTIR spectrum of pure starch exhibits characteristic peaks indicative of its chemical structure. The peak observed at approximately 3200–3600 cm^−1^ corresponds to the stretching vibrations of hydroxyl (OH) groups, which are abundant in starch molecules^9,76^. Another prominent peak around 2900–3000 cm^−1^ signifies the stretching vibrations of C–H stretching of the glucose units^77^. Additionally, a peak at about 1640–1660 cm^−1^ suggests the presence of bound water molecules, reflecting the hydrated nature of starch. Moreover, a peak in the region of 1020–1150 cm^−1^ is associated with the stretching vibrations of C–O–C glycosidic linkages, which are fundamental to the starch molecular structure. The FTIR spectrum of pure nanocellulose reveals characteristic peaks that reflect its cellulose-based composition. A strong peak at around 3300–3600 cm^−1^ corresponds to the stretching of hydroxyl (OH) groups abundant in cellulose. Peaks in the range of 2900–2905 cm^−1^ are attributed to the C–H stretching vibration within the cellulose backbone^78^. Additionally, peaks around 1150–1100 cm^−1^ indicate the presence of a C–O–C glycosidic bond and a C–C ring, which are key structural elements of cellulose molecules^79^. In the FTIR spectrum of the starch-nanocellulose composite film, characteristic peaks of both starch and nanocellulose components are typically observed. These peaks overlap, indicating that both materials were successfully incorporated into the composite matrix. The interpretation of the peaks is consistent with those observed in the spectra of pure starch and nanocellulose, confirming the presence of the respective functional groups in the composite film. When tea extract is incorporated into the starch-nanocellulose composite film, additional peaks may appear in the FTIR spectrum, reflecting the presence of compounds from the tea extract. In FTIR spectroscopy, the peak counts for bands associated with polyphenols, flavonoids and other phytochemicals present in the tea extract in starch films may vary depending on the specific functional groups in the compounds. These peaks could include bands associated with polyphenols, polysaccharides, and caffeine (theine) present in the tea extract^80^. This finding is consistent with the literature reported^10,38,47^. Polyphenols typically show peaks associated with OH groups around 3300–3500 cm^−1^, aromatic ring stretching around 1600–1700 cm^−1^ and C–O stretching around 1000–1300 cm^−1^. Flavonoids show peaks for C=C stretching in the aromatic ring around 1600–1700 cm^−1^ and C–O stretching around 1000–1300 cm^−1^ (Fig. 7a). The FTIR spectra of the different films after biodegradation under soil burial conditions showed different changes in the functional groups (Fig. 7b). The FTIR spectra of various films after biodegradation under soil burial condition showed different functional group changes. Imam et al.^81^ reported a decrease in OH absorbance (3100–3500 cm^−1^) due to starch degradation in starch-PVA-lignocellulosic fiber films after burial in soil for 120 days.

### EDX analysis

**Figure 8:**
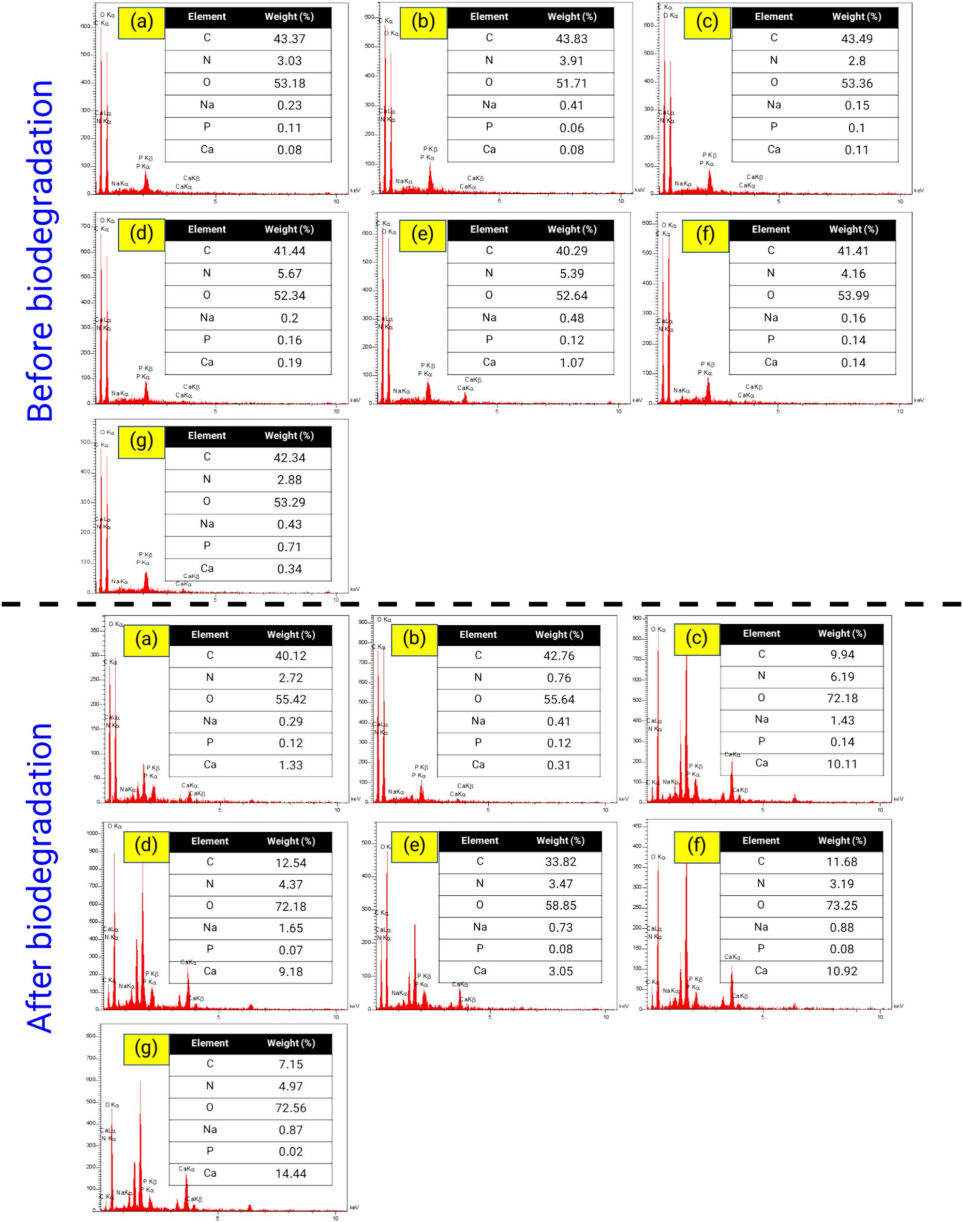
EDX spectrum of different films before and after biodegradation under soil burial conditions (30 days). (**a**) control (pure starch), (**b**) starch-2% nanocellulose, (**c**) starch-4% nanocellulose, (**d**) starch-6% nanocellulose, (**e**), starch-2% nanocellulose-tea extract, (**f**), starch-4% nanocellulose-tea extract, and (**g**) starch-6% nanocellulose-tea extract.

EDX analysis is a technique that can be used to analyze the elemental composition of a material. The main elements of the different films before and after biodegradation under burial conditions are shown in Fig. 8. The main composition consisted of C, N, O, Na, P, and Ca. These results clearly show that the carbon content in the sample decreased after the biodegradation process. The EDX analysis revealed a reduction in carbon content and an increase in oxygen content in the starch film sample that underwent degradation in the soil. This result may be caused by the processes of decomposition and destruction of starch by microorganisms and other biochemical processes in the soil. These processes can lead to ion exchange between carbon and oxygen ions with other ions and consequently to a decrease in carbon content and an increase in oxygen in the samples. In addition, bacteria employ low molecular weight polymer chains in the process of microbial digestion. These intermediates serve as energy sources for the microbes and undergo breakdown into compounds including water, CO_2_, and other metabolic biomass^55,72^. Similar EDX results have been obtained in earlier reports^82–85^.

### Effect of biodegradation process on soil properties

The ANOVA indicated a significant treatment (film type) and biodegradation time effect on the physical and chemical properties of soil under biodegradation (*p* < 0.01) (Table 3). In some treatments, such as moisture content, TOC, and pH, the interaction effect (treatment × time) of variable factors was not significant (*p* > 0.05).

**Table 3:** Analysis of variance (ANOVA) results for the effect of the biodegradation process on the physical and chemical properties of soil.

SOV	df	Mean square (MS)					
		Moisture content	Water holding capacity (WHC)	Soil respiration	Cold water extractable organic carbon (CWEOC)	Hot water extractable organic carbon (HWEOC)	Total organic carbon (TOC)
Treatment (A)	7	30.870**	501.737**	4151.079**	0.0001**	0.0001**	0.087**
Time (B)	2	2264.900**	1154.007**	21,697.01**	0.001**	0.001**	0.159**
A × B	14	0.430^ns^	23.549**	623.754**	2.655E−5**	4.856E−5**	0.006^ns^
Error	48	0.573	8.920	32.363	7.000E−5	4.893E−6	0.005

SOV	df	Mean square (MS)					
		Total nitrogen	Electrical conductivity (EC)	pH			
Treatment (A)	7	0.005**	0.099**	0.024^ns^			
Time (B)	2	0.027**	0.172**	0.225**			
A × B	14	0.003**	0.007*	0.002^ns^			
Error	48	1.618E−5	0.003	0.014			

**Significant at *p < 0.01; *significant at *p < 0.05.

### Moisture content and water holding capacity (WHC)

**Figure 9:**
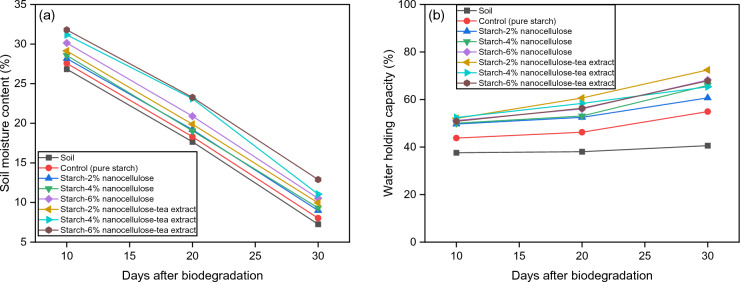
Effect of biodegradation process on (**a**) soil moisture content and (**b**) water holding capacity.

The results showed that the presence of films in the soil increased soil moisture content and WHC compared to soil without films (p < 0.05) (Fig. 9). The capacity of soil to retain water is crucial for plant development since water is essential for the synthesis of glucose in plants^86^. The increase in moisture content of soils with buried films compared to control soils can be attributed to several factors related to the degradation of the film material and its effects on soil properties. Nanocellulose usually demonstrates pronounced hydrophilicity as a result of the many hydroxyl groups (OH) or hydrophilic functional groups that are included during the manufacturing process^87^. This strong hydrophilic property causes more moisture absorption and increases soil moisture content and WHC. When biodegradable films are placed in soil, they create a small environment that helps to retain moisture. This occurs by reducing evaporation and enhancing water holding. The film material acts as a barrier that limits water loss from the soil surface. This leads to higher moisture levels in the area surrounding the buried samples. As the film material decomposes, it releases organic matter into the soil, which helps to improve soil structure and porosity. This ultimately leads to an increase in the soil's ability to retain water. Additionally, the breakdown of the film material stimulates microbial activity in the soil, which can further impact soil moisture levels. Therefore, the observed increase in soil moisture levels is a result of reduced evaporation, improved soil structure, and increased microbial activity caused by the decomposition of the film material. Earlier reports showed that the starch-based films can enhance the soil's water holding capacity or moisture content. For instance, Nassaj-Bokharaei, et al.^86^ found that the reinforced starch-based hydrogels with natural char nanoparticles, significantly improved soil WHC, nutritional indices (16–29% increase), and tomato plant growth (22–45% increase). Similarly, Sen and Das^88^ reported that biodegradable films made from starch and polyvinyl alcohol (PVA) increased soil WHC (27.5%), improving soil quality and supporting plant growth conditions.

### Soil respiration

**Figure 10:**
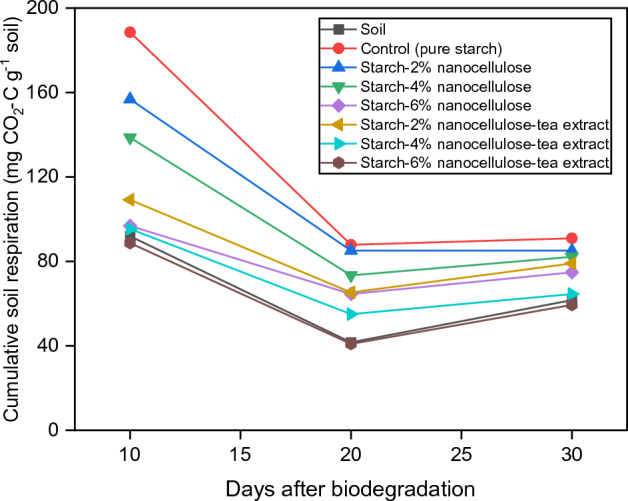
Effect of biodegradation process on soil respiration.

The highest microbial respiration was observed on the 10th day after the films were applied to the soil. This was related to the treatment of pure starch (188.53 mg CO_2_–C g^−1^ soil), which was to be expected. SNBTE films had lower soil respiration compared to starch-nanocellulose films within 10–30 days after addition to the soil, regardless of their concentration (Fig. 10). This phenomenon arises from the fact that it serves as a more readily available carbon source for soil microorganisms, leading to the production of a diverse array of starch-degrading enzymes^9^. Compared to pure starch films, starch-nanocellulose films can hinder the access of microbes to food sources and suitable conditions for their growth below the soil surface, leading to a decrease in microbial activity below the soil surface. Antibacterial and antifungal effects of active ingredients in black tea extracts can reduce microbial activity^43,44^. In addition, the physical and structural properties of films containing nanocellulose, compared to pure starch films, can reduce the permeability to air and moisture, which in turn limits the access of microbes to food sources and suitable conditions for their growth below the soil surface. This can lead to a decrease in microbial activity below the soil surface. Soil respiration rates in soils containing buried film samples are higher than those in control soils due to several interrelated factors. Biodegradable films in soil become a substrate for microbial colonization and activity, leading to an increase in soil respiration rates as the microorganisms degrade the organic components of the film material. The presence of film material also stimulates the growth and activity of microbial biomass, which further increases soil respiration rates. In addition, the degradation of the film material leads to changes in the physicochemical properties of the soil, which can influence the microbial respiration processes. This increase in soil respiration is the result of the combined effects of increased substrate availability, microbial biomass growth, and altered soil conditions. This fact is well supported by the finding of Malekzadeh et al.^9^ and Nassaj-Bokharaei et al.^86^.

### Carbon analysis

The data presented in Table 4 show the biodegradation of the different film types under soil burial conditions at different time intervals (10, 20, and 30 days). In terms of CWEOC, the data show that the control group (pure starch) has higher values than soil alone across all time points. Films with nanocellulose, both alone and with tea extract, generally have lower CWEOC values than the control group, suggesting a potential reduction in the leaching of organic carbon into the soil by these film compositions. Interestingly, the addition of tea extract appears to have a slightly decreasing effect on CWEOC values, which is particularly noticeable for the 4 and 6% nanocellulose compositions. The HWEOC values generally follow a similar trend, with the control group showing consistently higher values compared to the pure soil. Films containing nanocellulose generally had lower HWEOC values compared to the control group, indicating a potential reduction in the release of water-soluble organic carbon into the soil by these film compositions. Again, the addition of tea extract appears to have a slightly decreasing effect on HWEOC content, particularly pronounced with the 2% nanocellulose composition. Analysis of the TOC content shows a similar pattern, with the control group consistently showing higher values than the soil alone. Films containing nanocellulose generally have higher TOC values than the control group, indicating a possible increase in the total organic carbon content in the soil due to the decomposition of these films. The addition of tea extract generally shows a mixed effect on TOC content, with effects varying depending on nanocellulose concentration.

**Table 4:** Effect of film biodegradation on soil carbon.

Film type	Days after biodegradation under soil burial condition		
	10	20	30
Cold water extractable organic carbon (CWEOC), %			
Soil	0.010 ± 0.0010	0.0110 ± 0.0010	0.0143 ± 0.0021
Control (pure starch)	0.016 ± 0.0010	0.0243 ± 0.0015	0.0363 ± 0.0023
Starch-2% nanocellulose	0.015 ± 0.0010	0.0203 ± 0.0040	0.0333 ± 0.0015
Starch-4% nanocellulose	0.014 ± 0.0015	0.0190 ± 0.0026	0.0283 ± 0.0041
Starch-6% nanocellulose	0.014 ± 0.0010	0.0183 ± 0.0056	0.0290 ± 0.0034
Starch-2% nanocellulose-tea extract	0.012 ± 0.0030	0.0176 ± 0.0025	0.0263 ± 0.0040
Starch-4% nanocellulose-tea extract	0.013 ± 0.0010	0.017 ± 0.0052	0.020 ± 0.0005
Starch-6% nanocellulose-tea extract	0.012 ± 0.0015	0.014 ± 0.0015	0.018 ± 0.0011
Hot water extractable organic carbon (HWEOC), %			
Soil	0.013 ± 0.0015	0.0195 ± 0.0035	0.0257 ± 0.0041
Control (pure starch)	0.016 ± 0.0010	0.0202 ± 0.0033	0.0307 ± 0.0029
Starch-2% nanocellulose	0.011 ± 0.0013	0.0156 ± 0.0027	0.0257 ± 0.0027
Starch-4% nanocellulose	0.018 ± 0.0019	0.0312 ± 0.0039	0.0434 ± 0.0041
Starch-6% nanocellulose	0.011 ± 0.0009	0.0138 ± 0.0014	0.0228 ± 0.0004
Starch-2% nanocellulose-tea extract	0.010 ± 0.0001	0.0130 ± 0.0020	0.0138 ± 0.0005
Starch-4% nanocellulose-tea extract	0.010 ± 0.0004	0.0121 ± 0.0012	0.0130 ± 0.0007
Starch-6% nanocellulose-tea extract	0.010 ± 0.0007	0.0118 ± 0.0005	0.0127 ± 0.0007
Total organic carbon (TOC), %			
Soil	0.37 ± 0.0200	0.38 ± 0.0321	0.40 ± 0.0608
Control (pure starch)	0.44 ± 0.0608	0.44 ± 0.0152	0.66 ± 0.0152
Starch-2% nanocellulose	0.46 ± 0.0503	0.49 ± 0.1150	0.67 ± 0.0709
Starch-4% nanocellulose	0.51 ± 0.0721	0.54 ± 0.0793	0.69 ± 0.0929
Starch-6% nanocellulose	0.55 ± 0.0814	0.63 ± 0.0152	0.71 ± 0.1250
Starch-2% nanocellulose-tea extract	0.52 ± 0.0702	0.58 ± 0.0750	0.69 ± 0.0556
Starch-4% nanocellulose-tea extract	0.59 ± 0.1342	0.68 ± 0.0416	0.72 ± 0.0776
Starch-6% nanocellulose-tea extract	0.61 ± 0.0854	0.70 ± 0.0360	0.78 ± 0.0529

Values represent the mean ± standard deviation.

The increase in CWEOC and HWEOC of soils containing buried starch/nanocellulose film samples compared to control soils can be explained by several factors related to the degradation of the film material and its subsequent interaction with the soil environment. Starch-nanocellulose films undergo biodegradation processes after burial in the soil, which are promoted by microorganisms in the soil. The presence of starch in films can increase microbial activity and diversity in the soil, as these materials can serve as substrates for the growth of soil microflora^88^. As these films decompose, organic carbon compounds are released into the surrounding soil matrix. Starch, a polysaccharide, is enzymatically hydrolyzed by soil microbes, resulting in the release of soluble sugars and other organic compounds. Similarly, nanocellulose, a carbohydrate-based material, also contributes to the organic carbon pool during decomposition. The presence of these organic compounds increases the CWEOC and HWEOC content in the soil. In addition, the degradation of starch-nanocellulose films can stimulate microbial activity, leading to an increase in microbial biomass and metabolic processes, which in turn contribute to the release of organic carbon into the soil solution. In addition, the physical structure of the film material can promote soil aggregation and improve soil porosity, which facilitates the penetration of water and organic carbon compounds, thereby increasing the CWEOC and HWEOC content. The observed increase in CWEOC and HWEOC content in soils containing buried starch-nanocellulose film samples compared to control soils is therefore a consequence of the complex interactions between the degrading film material, soil microorganisms and soil organic carbon dynamics. In a similar study, Sen and Das^88^ reported that after 35 days of burying starch-based antibacterial films in the soil, the amount of carbon increased by 14.4% compared to the control treatment. Li et al.^89^ in the study on the biodegradability of starch, polylactic acid and cellulose-based mulches on soil quality, found that these mulches increase the amount of organic carbon in the soil, but their changes are very small and depend on the production system and incubation time.

### Total nitrogen

**Figure 11:**
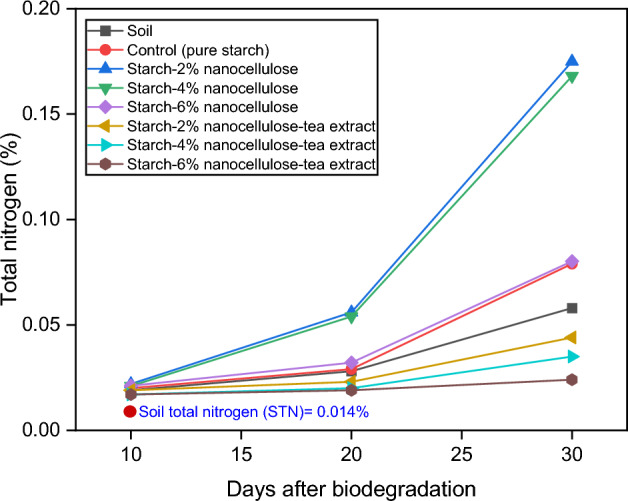
Effect of biodegradation process on soil total nitrogen.

Figure 11 shows the total nitrogen content in the soil samples and the different films at different time intervals (10, 20, and 30 days) after biodegradation under soil burial conditions. Considering solely the total nitrogen content in the soil, it appears to remain relatively stable over time, indicating minimal variations in nitrogen levels under the specified conditions. In contrast, the total nitrogen content in the control group (pure starch) and in the different films generally increases over time, indicating a release or accumulation of nitrogen during the biodegradation process.

Comparing the different film compositions, those containing nanocellulose showed a higher total nitrogen content than the control group at all time points, indicating a possible contribution of nanocellulose to nitrogen availability in the soil during biodegradation. Interestingly, the addition of tea extract along with nanocellulose appears to have a mitigating effect on total nitrogen content, with lower values observed compared to nanocellulose-only compositions, particularly noticeable at the 4% and 6% nanocellulose concentrations. The increase in total nitrogen content of soils containing buried starch-nanocellulose film samples compared to control soils can be attributed to several mechanisms related to the decomposition of the film material and its interactions with the soil ecosystem. Starch nanocellulose films, when buried in the soil, are degraded by microorganisms in the soil. During this process, the organic components of the film material, including starch and cellulose, are broken down into simpler compounds, some of which contain nitrogen, such as amino acids and proteins. The decomposition of the film material releases nitrogen into the soil matrix, which contributes to an increase in the total nitrogen content. In addition, the presence of starch and nanocellulose in the film material can increase microbial activity in the soil, leading to increased nitrogen mineralization and turnover of organic nitrogen compounds. In addition, the degradation of the film material can change the physical properties of the soil, such as porosity and water retention, which can affect nitrogen availability and cycling processes. The increase in total nitrogen observed in soils with buried starch nanocellulose film samples compared to control soils is therefore a consequence of interactions between the decomposing film material, soil microorganisms and nitrogen dynamics in the soil. Sen and Das^88^ reported that the amount of available nitrogen and total nitrogen in the soil containing starch-based antibacterial films was 86% and 157% higher than the control, respectively. In another study, Zhao et al.^90^ reported that the biodegradable films enhanced soil nitrogen levels but decreased organic carbon content, affecting the C/N ratio. Koskei et al.^91^ found that biodegradable residual films in agriculture improved soil nitrogen content and reduced the C/N ratio.

### Electrical conductivity (EC) and pH

**Figure 12:**
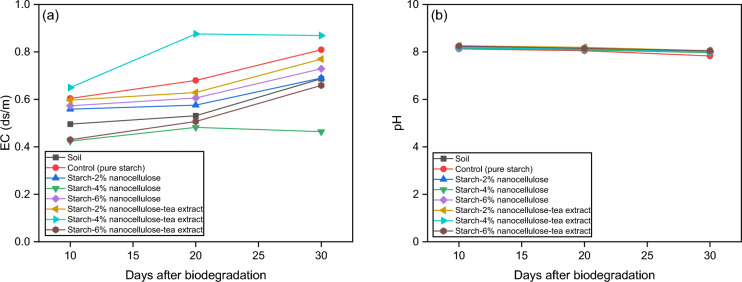
Effect of biodegradation process on (**a**) EC and (**b**) pH.

Figure 12 shows the effects of biodegradation of the film on the electrical conductivity (EC) and pH of the soil over 30 days. As for pH, slight variations were observed between the different film compositions and over time. The control group and the film compositions containing tea extract tended to have slightly lower pH values than the soil alone and the other film compositions, indicating a potential acidifying effect. Conversely, film compositions with higher concentrations of nanocellulose show pH values closer to those of the soil alone, indicating less influence on soil acidity. The decrease in pH might have been caused by the decomposition of the buried film's organic matter by soil microorganisms, which produce organic acids^88^. In addition, the degradation of starch-nanocellulose films can also influence soil pH dynamics. Initially, the decomposition of organic matter from the film material can release acidic compounds into the soil, resulting in a temporary decrease in pH. However, as decomposition progresses, the release of organic acids can be balanced by the accumulation of alkaline substances such as carbonates and bicarbonates produced by microbial activity. This shift towards alkalinity can contribute to an increase in soil pH over time.

When analyzing the EC values, fluctuations can be observed over time and with the different film compositions. In general, the control group (pure starch) and the film compositions with tea extract tend to have higher EC values than the soil alone and the other film compositions. Conversely, films with higher concentrations of nanocellulose (4% and 6%) have lower EC values, indicating a potential mitigating effect on soil salinity. The increase in EC of soils containing buried starch nanocellulose film samples compared to control soils can be attributed to several interrelated mechanisms related to the decomposition of the film material and its interaction with the soil environment. Starch nanocellulose films, when buried in soil, undergo microbial degradation, which is promoted by soil microorganisms. During this process, the organic components of the film, such as starch and cellulose, are broken down into simpler compounds, releasing ions and organic matter into the soil matrix. This organic matter contributes to the soil's nutrient pool and can increase microbial activity, leading to increased mineralization of organic compounds and subsequent release of ions, including those that contribute to EC, such as potassium, calcium and magnesium. Sen and Das^88^ reported a 10.5% decrease in soil pH as a result of buried starch-based antibacterial films. Qi et al.^92^ reported that after 4 months, soil pH values ​​decreased in treatments containing 1% bio-macroplastic compared to the control. But soil EC has been increasing. Li et al.^89^ reported substantial changes in soil EC and pH, suggesting that cellulose and starch-based mulch films significantly impact these soil properties.

### MDW and GMD

The provided data in Table 5 presents the effect of the biodegradation process on MDW and GMD after 30 days. The higher the MWD and GMD values, the better the water-stability of the soil aggregate. On the other hand, the lower the values, the worse the water-stability^90^. Analyzing MDW, the control group (pure starch) exhibits an MDW value of 0.035 mm, slightly higher than that of the soil alone, which has an MDW of 0.024 mm. The film compositions containing nanocellulose, either alone or with tea extract, generally show slightly lower MDW values compared to the control group, indicating a potential influence of these film compositions on the aggregation of soil particles. However, the differences are relatively small, suggesting that the addition of nanocellulose and tea extract may have a limited impact on the stability and size of soil aggregates. Examining GMD, similar trends are observed, with the control group displaying a GMD of 1.130 mm, slightly higher than the soil alone, which has a GMD of 1.096 mm.

**Table 5:** Effect of biodegradation process on MDW and GMD after 30 days.

Film type	MDW (mm)	GMD (mm)
Soil	0.024 ± 0.006	1.096 ± 0.026
Control (pure starch)	0.035 ± 0.001	1.130 ± 0.007
Starch-2% nanocellulose	0.033 ± 0.003	1.118 ± 0.001
Starch-4% nanocellulose	0.033 ± 0.003	1.125 ± 0.002
Starch-6% nanocellulose	0.037 ± 0.002	1.131 ± 0.004
Starch-2% nanocellulose-tea extract	0.041 ± 0.001	1.146 ± 0.003
Starch-4% nanocellulose-tea extract	0.046 ± 0.003	1.164 ± 0.005
Starch-6% nanocellulose-tea extract	0.044 ± 0.001	1.157 ± 0.002

Film compositions containing nanocellulose, both alone and with tea extract, generally exhibit GMD values comparable to or slightly higher than the control group, indicating a potential influence of these compositions on the overall size distribution of soil aggregates. However, once again, the differences are relatively minor, suggesting that the addition of nanocellulose and tea extract may have a limited effect on the geometric mean diameter of soil aggregates. A possible explanation for the increase in the MDW and GMD after the biodegradation of SNBTE films can be attributed to the physical and chemical effects of these substances in the decomposition process and the changes they cause in the soil structure. The presence of nanocellulose and tea extract in the films likely leads to an increase in the density and stability of soil aggregates, because these materials can act as adhesion agents and bind soil particles together, which can lead to the creation of larger and more stable soil aggregates that they are created by the decomposition of films. In previous studies, microplastics were also shown to reduce macroaggregate fractions and alter water-stable aggregate profiles^93^. Zhao et al.^90^ reported that films with biodegradable and plastic residues applied at high levels reduced MWD and GMD. This means that the films prevent the formation of macro-soil aggregates (> 0.25 mm). The difference of this result compared to the results obtained in this research can be caused by the origin of the film used.

### Policy implications

The investigation into the biodegradation behavior of SNBTE films under soil burial conditions and their impact on soil physical and chemical properties suggests several policy implications. Firstly, there's a need for comprehensive environmental impact assessments to evaluate the suitability of these biodegradable films in agriculture, considering their interaction with soil and potential effects on soil quality. Regulatory frameworks should be established or updated to ensure the responsible use of biodegradable materials, incorporating standards for film composition and degradation rate to protect soil health. Governments and agricultural agencies can encourage the adoption of sustainable agriculture practices by promoting the use of biodegradable films, thus reducing plastic pollution and preserving soil fertility. Additionally, increased funding for research and development can drive innovation in biodegradable film technology, leading to more environmentally friendly alternatives. Education and awareness campaigns are essential to inform stakeholders about the benefits and proper application of biodegradable films, fostering a more sustainable agricultural sector.

### Limitations, challenges, and prospects of research

The study of the biodegradation behavior of SNBTE films under soil burial conditions provides both challenges and opportunities for the development of sustainable packaging materials. By replacing conventional plastic films, this film system may offer a new and biodegradable material for food packaging and other applications. However, there are several limitations and challenges in the study of biodegradable packaging materials. Firstly, the biodegradation process is complex, and many factors can influence it, including soil composition, potential microbial activity, and environmental conditions. Accurately assessing the kinetics and mechanisms of biodegradation of composite films is therefore a big challenge. Secondly, the biodegradation rates of various components in the composite films can be different. Particularly for nanocellulose, the biodegradation mechanism of this component is not clear, which may change the biodegradability of the overall film, as well as the dynamics of nutrient release. In addition, the potential impact of the degradation by-products of composite films on soil respiration and nutrient availability also requires careful evaluation to verify the compatibility of the materials with the surrounding environment. In conclusion, this study takes a step towards the development of sustainable packaging materials by exploring the biodegradation behavior of unique composite films. Additionally, our work provides strategies to optimize the biodegradable behavior of composite films and further enhance their performance in environmental applications. The knowledge gained in this study is crucial for the development of formulations that are not only biodegradable but also have a low environmental impact. This will help to move the packaging industry one step closer to a more sustainable path. However, the development and evaluation of biodegradable composite films also require close collaboration between material scientists, microbiologists, and environmental engineers. Interdisciplinary cooperation is essential for the sustainable development of biodegradable composite films. The problem-solving process in this area requires considerable support from all areas.

## Conclusions

The study concludes that SNBTE films biodegrade effectively under soil burial conditions, demonstrating their potential as an environmentally friendly alternative to conventional plastics. The incorporation of 6.0% nanocellulose managed to improve the tensile strength up to 10.54 MPa by 40.4% compared to pure starch film. The addition of tea extract to the films significantly decreased their biodegradation rate, which is due to the polyphenolic compounds that are likely to stimulate microbial activity. The degradation of these films had a positive effect on soil health by increasing microbial activity and organic matter content, while pH and EC remained stable. These results underline the dual benefit of using SNBTE films, i.e. reducing plastic waste and improving soil quality. Therefore, these biodegradable films are promising for use in sustainable packaging and agriculture, contributing to both waste management and improved soil health.

## Data Availability

The data provided in this study is available with the corresponding author and can be presented on considerable request.
